# Relationships between quantitative retinal microvascular characteristics and cognitive function based on automated artificial intelligence measurements

**DOI:** 10.3389/fcell.2023.1174984

**Published:** 2023-06-21

**Authors:** Xu Han Shi, Li Dong, Rui Heng Zhang, Deng Ji Zhou, Sai Guang Ling, Lei Shao, Yan Ni Yan, Ya Xing Wang, Wen Bin Wei

**Affiliations:** ^1^ Beijing Tongren Eye Center, Beijing Key Laboratory of Intraocular Tumor Diagnosis and Treatment, Beijing Tongren Hospital, Capital Medical University, Beijing, China; ^2^ Beijing Ophthalmology and Visual Sciences Key Lab, Beijing Tongren Hospital, Capital Medical University, Beijing, China; ^3^ Medical Artificial Intelligence Research and Verification Key Laboratory of the Ministry of Industry and Information Technology, Beijing Tongren Hospital, Capital Medical University, Beijing, China; ^4^ EVision Technology (Beijing) Co., Ltd., Beijing, China; ^5^ Beijing Ophthalmology and Visual Science Key Laboratory, Beijing Tongren Eye Center, Beijing Tongren Hospital, Beijing Institute of Ophthalmology, Capital Medical University, Beijing, China

**Keywords:** artificial intelligence, deep learning, retinal vascular, cognitive function, cognitive impairment

## Abstract

**Introduction:** The purpose of this study is to assess the relationship between retinal vascular characteristics and cognitive function using artificial intelligence techniques to obtain fully automated quantitative measurements of retinal vascular morphological parameters.

**Methods:** A deep learning-based semantic segmentation network ResNet101-UNet was used to construct a vascular segmentation model for fully automated quantitative measurement of retinal vascular parameters on fundus photographs. Retinal photographs centered on the optic disc of 3107 participants (aged 50–93 years) from the Beijing Eye Study 2011, a population-based cross-sectional study, were analyzed. The main parameters included the retinal vascular branching angle, vascular fractal dimension, vascular diameter, vascular tortuosity, and vascular density. Cognitive function was assessed using the Mini-Mental State Examination (MMSE).

**Results:** The results showed that the mean MMSE score was 26.34 ± 3.64 (median: 27; range: 2–30). Among the participants, 414 (13.3%) were classified as having cognitive impairment (MMSE score < 24), 296 (9.5%) were classified as mild cognitive impairment (MMSE: 19–23), 98 (3.2%) were classified as moderate cognitive impairment (MMSE: 10–18), and 20 (0.6%) were classified as severe cognitive impairment (MMSE < 10). Compared with the normal cognitive function group, the retinal venular average diameter was significantly larger (*p* = 0.013), and the retinal vascular fractal dimension and vascular density were significantly smaller (both *p* < 0.001) in the mild cognitive impairment group. The retinal arteriole-to-venular ratio (*p* = 0.003) and vascular fractal dimension (*p* = 0.033) were significantly decreased in the severe cognitive impairment group compared to the mild cognitive impairment group. In the multivariate analysis, better cognition (i.e., higher MMSE score) was significantly associated with higher retinal vascular fractal dimension (b = 0.134, *p* = 0.043) and higher retinal vascular density (b = 0.152, *p* = 0.023) after adjustment for age, best corrected visual acuity (BCVA) (logMAR) and education level.

**Discussion:** In conclusion, our findings derived from an artificial intelligence-based fully automated retinal vascular parameter measurement method showed that several retinal vascular morphological parameters were correlated with cognitive impairment. The decrease in retinal vascular fractal dimension and decreased vascular density may serve as candidate biomarkers for early identification of cognitive impairment. The observed reduction in the retinal arteriole-to-venular ratio occurs in the late stages of cognitive impairment.

## Introduction

Cognitive impairment is the most common neurodegenerative disorder. Severe cognitive impairment that leads to Alzheimer’s disease and dementia ultimately manifests as an extensive loss of cognitive ability and imposes a tremendous burden on patients, economies, healthcare systems, and society (Prince et al., 2013; Alzheimer’s Association, 2022). The prevalence of cognitive impairment is high globally, its pathogenesis is complex, and there are no effective treatments currently available. However, evidence-based preventative methods to delay the development and progression of the disease have been proposed. Therefore, it is particularly important to diagnose and prevent cognitive impairments at an early stage (Yu et al., 2020). Currently, the diagnosis of cognitive impairment relies on the detection of serum and protein biomarkers, examination of cerebrospinal fluid (CSF), and positron emission tomography (PET) scans. As a result of their high cost, high level of risk to patients posed by invasive procedures, high level of technical difficulty, and considerable time costs, these diagnostic procedures are not appropriate for early large-scale screening of the disease (Jack et al., 2018; Polanco et al., 2018; Fish et al., 2019). Consequently, it is essential to discover effective, noninvasive, easy-to-implement, and cost-efficient biomarkers that can identify individuals with cognitive impairment in its early stages to allow timely interventions to prevent or delay the onset of dementia.

Vascular diseases are a risk factor for cognitive impairment (Reitz et al., 2011; Ngolab et al., 2019). Previous studies have demonstrated that vascular risk factors affecting the cerebral microcirculation may also play a significant role in cognitive impairment (Czakó et al., 2020; Sur et al., 2020). In the majority of cases characterized by cognitive impairment, variations in cerebral microvascular characteristics, such as increased tortuosity and arteriolar narrowing, and their association with degenerative changes have been reported (Liesz, 2019; O'Neill et al., 2021). There are many similarities between the retina and the brain, including their physiological characteristics, embryological origins, cellular resemblances, precise neuron cell layers, and microvasculature (Xie et al., 2023). As a window to the brain, the retina offers an excellent opportunity for researchers to investigate the pathogenesis of numerous ophthalmic and neurodegenerative diseases (Snyder et al., 2021; Zhang et al., 2021). Accumulating reports of retinal imaging utilizing various imaging techniques have revealed a correlation between retinal vascular alterations and the incidence of cognitive impairment (Patton et al., 2005; Ravi Teja et al., 2017; Ngolab et al., 2019; Wu et al., 2020). However, it has been determined that some of the findings of these reports are inconsistent, and so the relationship between retinal vascular parameters (e.g., retinal vascular diameter, retinal vascular tortuosity, etc.) and cognitive function remains controversial. We therefore conducted a study to further clarify the relationship between retinal vascular parameters and cognitive function.

Retinal fundus photography is a valuable technique for the quick assessment of retinal vascular characteristics. In the last two decades, computer programs developed for medical imaging have made it possible to perform a number of computer-based measurements on retinal fundus photography and ultimately demonstrate a relationship between retinal vascular changes and clinical characteristics (Cheung et al., 2011a; Dervenis et al., 2019). With the help of computer-assisted analysis programs, characteristics of the retinal vasculature, including fractal dimension, tortuosity, and vessel caliber, can be assessed quantitatively (Cheung et al., 2011b). Nevertheless, until recently, the majority of quantitative measurements have been carried out with the assistance of semiautomated retinal vessel measurements software, such as SIVA, IVAN, and VAMPIRE (Trucco et al., 2015; Chan et al., 2017; Czakó et al., 2020). These methods involve manual input and adjustment by qualified technicians, are time-consuming and prone to error, and are therefore inefficient (McGrory et al., 2018; Mautuit et al., 2022).

In recent years, deep learning algorithms have proven superior performance in assessing diabetic retinopathy and other retinal characteristics (Ting et al., 2017; Dong et al., 2022). In this study, we developed a deep learning model to perform fully automated segmentation of retinal vessels and quantitatively evaluate retinal vessel parameters, including retinal vessel diameter, retinal vessel curvature, retinal vessel fractal dimension, retinal vessel density, etc. We described retinal vascular characteristics that may serve as candidate biomarkers for the early identification of cognitive impairment as well as for the progression of the disease.

## Materials and methods

### Study population

The Beijing Eye Study 2011 is a cross-sectional population-based study that was conducted in five communities in the urban district in northern central Beijing and in three communities in the village area in southern Beijing. A detailed description of the population and the design of the study has been provided previously (Liu et al., 2010; Yan et al., 2015). Among 4,403 eligible individuals, 3,468 individuals [response rate: 78.8%; females: 1,963 (56.6%); mean age: 64.6 ± 9.8 years; median age: 64 years; age range: 50–93 years] participated in the study. The Medical Ethics Committee of Beijing Tongren Hospital approved the study protocol, and all participants gave informed written consent in accordance with the Declaration of Helsinki. The ethics committee confirmed that all methods were performed in accordance with the relevant guidelines and regulations.

### Ophthalmic and general examinations

All examinations were conducted in the communities at either schoolhouses or community houses. Participants in the study were interviewed by trained research technicians and completed standardized questionnaires. The interview included standardized questions on demographics and socioeconomic factors, such as age, sex, education level, current smoking status, and systemic disease histories, such as arterial hypertension, diabetes mellitus, cardiovascular disease, and infarction (including both myocardial infarction and brain infarction). The education levels of participants were classified as “illiteracy,” “partial illiteracy with knowledge of some Chinese words,” “primary school education,” “middle school education,” and “college or higher education.”

The ophthalmic examination included measurement of best corrected visual acuity (BCVA) (logMAR), slit lamp-assisted biomicroscopy of the anterior segment of the eye, and fundus photographs centered on the optic disc (nonstereoscopic photograph of 45° of the central fundus; fundus camera type CR6-45NM; Canon Inc., Tokyo, Japan).

Cognitive function was assessed as a cognitive function score using the Mini-Mental State Examination (MMSE) (Folstein et al., 1975; Crum et al., 1993). The MMSE is a widely used and validated screening tool for detecting cognitive impairment. In clinical practice, it exhibits moderate to high sensitivity and specificity. It is particularly useful when comparing individuals across a wide age range (Folstein et al., 1975; Tombaugh and McIntyre, 1992). Cognitive impairment was defined as an MMSE score < 24, in line with previous studies that have demonstrated good sensitivity and specificity at this cut point. Further grading of cognitive impairment was performed. Mild cognitive impairment was defined as an MMSE score ranging between 19 and 23 points, moderate cognitive impairment was defined as an MMSE score ranging between 10 and 18 points, and severe cognitive impairment was defined as an MMSE score < 10 (Folstein et al., 1975; Tombaugh and McIntyre, 1992; Liew et al., 2009; Jonas et al., 2018).

**FIGURE 1:**
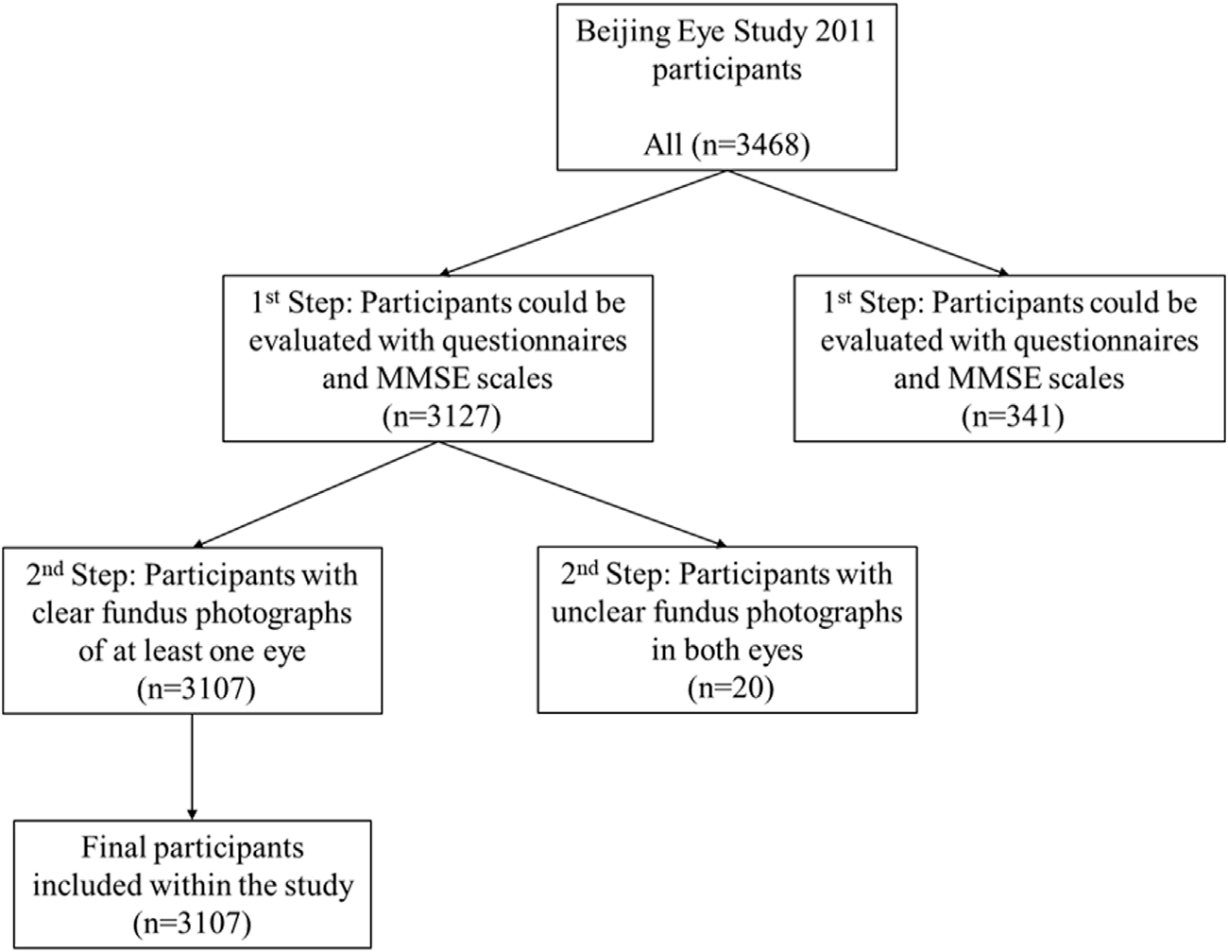
A flow chart of participant inclusion and exclusion criteria.

A patient was included if he or she could be evaluated with questionnaires and MMSE scales. The exclusion criteria were as follows: unclear bilateral fundus photographs of the eyes (inability to clearly visualize the optic disc and retinal vasculature) that could not be analyzed; and inability to evaluate the patient with questionnaires and MMSE scales (Figure 1). The data of all the right eyes were included in the current study. If a clear fundus photograph of the right eye could not be obtained, the data from the left eye were included.

### Quantitative measurements of retinal vascular parameters based on artificial intelligence automatic analysis technology

Participants’ color fundus photographs centered on the optic disc were analyzed. Included in the measurements were the retinal vascular branching angle, vascular fractal dimension, vascular average diameter, vascular average tortuosity, and retinal vascular density; the vascular average diameter and vascular average tortuosity were analyzed further in the annular regions 0.5–1.0 papillary diameter (PD) (C1), 1.0–1.5 PD (C2), 1.5–2.0 PD (C3), and 2.0–2.5 PD (C4) from the optic disc border. The vascular fractal dimension indicates the branching complexity of the retinal vascular network, reflecting the distribution of blood throughout the entire retinal circulation, with larger values indicating branching complexity (Liew et al., 2008). The retinal vascular average tortuosity indicates the degree of bend in retinal vessels. A smaller value indicates a flatter retinal vessel (Vilela et al., 2021). The annular area close to the optic disc provides a better measure of the diameter of the central retinal arterioles and venules. The annular region further from the optic disc, where there are more retinal vascular branches, can better demonstrate the branching complexity of the retinal vascular network and the degree of curvature of the retinal vessels.

In this study, we developed a computer image processing method using deep learning and computer vision technology to automatically segment retinal vessels and optic disc features on color fundus images based on the principle of human visual bionics and then extracted the centerline of blood vessels. Through the fusion of deep learning and computer vision technology, morphological parameters such as vascular diameter, vascular tortuosity, vascular fractal dimension, vascular branching angle, and vascular density can be calculated.

### Preprocessing of images

**FIGURE 2:**
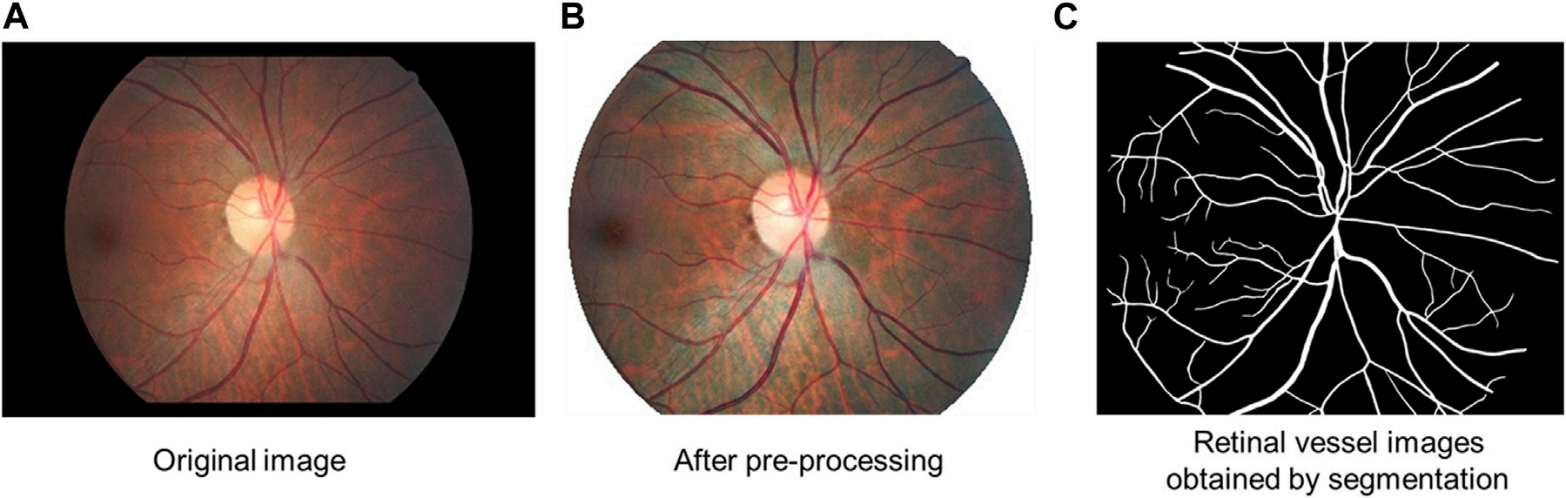
Diagram of retinal vessel segmentation. **(A)** Original image. **(B)** Image after pre-processing: regions of interest (ROI) were extracted, denoised, normalized, and enhanced. **(C)** Retinal vessel images obtained by segmentation.

To enhance the fundus images, regions of interest (ROI) were extracted, denoised, normalized, and enhanced (Xu et al., 2019; Shao et al., 2021). After the channel separation of the image, the threshold segmentation method was used on the red (R) channel to obtain the preselected ROI. The threshold for the ROI preselection area was used as 1/3 of the average grey value of the R channel. Afterward, the preselected area was filtered based on its location, area, roundness, and other attributes. In order to filter the area, we selected the largest area in the ROI preselection area. Then, the boundary was determined using morphological operations (image opening operations) to obtain the final ROI. On the color fundus image, this area represented the effective fundus retinal imaging area, which reduced the interference of invalid areas such as the background on the subsequent feature recognition and segmentation process. In the following step, the noise was removed by applying a low-pass filter (median filtering) to reduce the noise resulting from the camera’s imaging procedures. Furthermore, the color, brightness, and size of the images were normalized by mean calibration and resampling to minimize the variability between images. Mean calibration means adjusting the mean value of brightness and color of each image to the mean of the statistical values of all images. Resampling is the linear difference method used to scale all images to a uniform size. Additionally, the images were enhanced using the contrast-limited adaptive histogram equalization (CLAHE) algorithm, which enhanced the retinal features on the images (Figure 2B).

### Segmentation of retinal vessels

In this study, we use a deep learning-based semantic segmentation network ResNet101-UNet to construct a retinal vessel segmentation model, which adopts a cross-layer connectivity architecture and is capable of extracting vessel features at different scales (Ronneberger et al., 2015). A semiautomatic machine-assisted annotation method is employed to annotate the sample. First, the color image is converted into grayscale according to the following formula.

Gray=R×0.299+G×0.587+B×0.144

**FIGURE 3:**
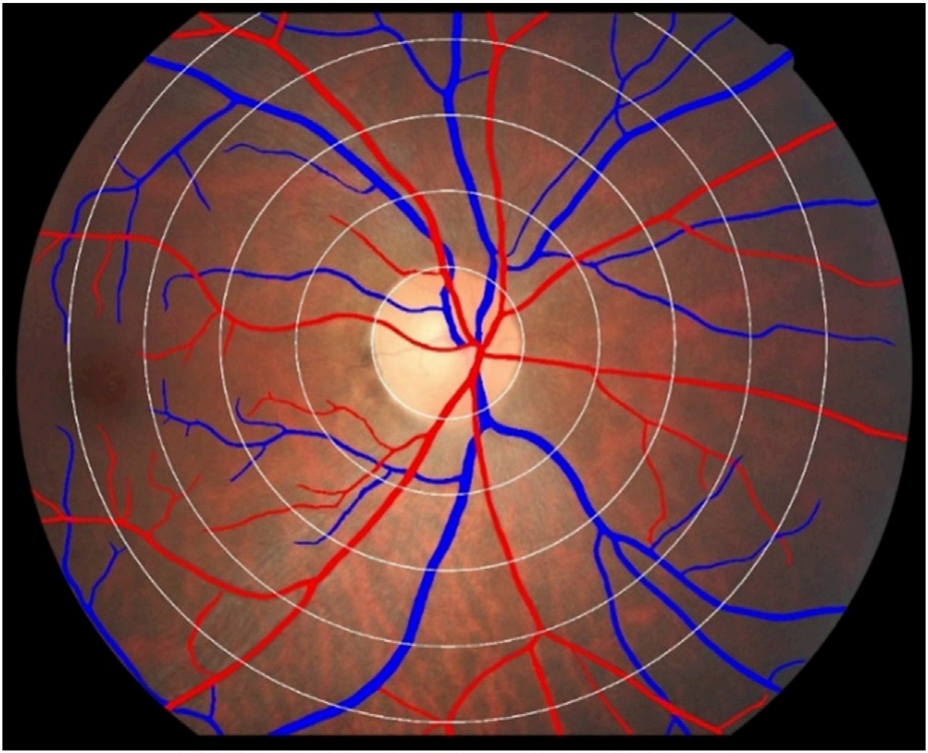
Identification of retinal arteries and veins.

**FIGURE 4:**
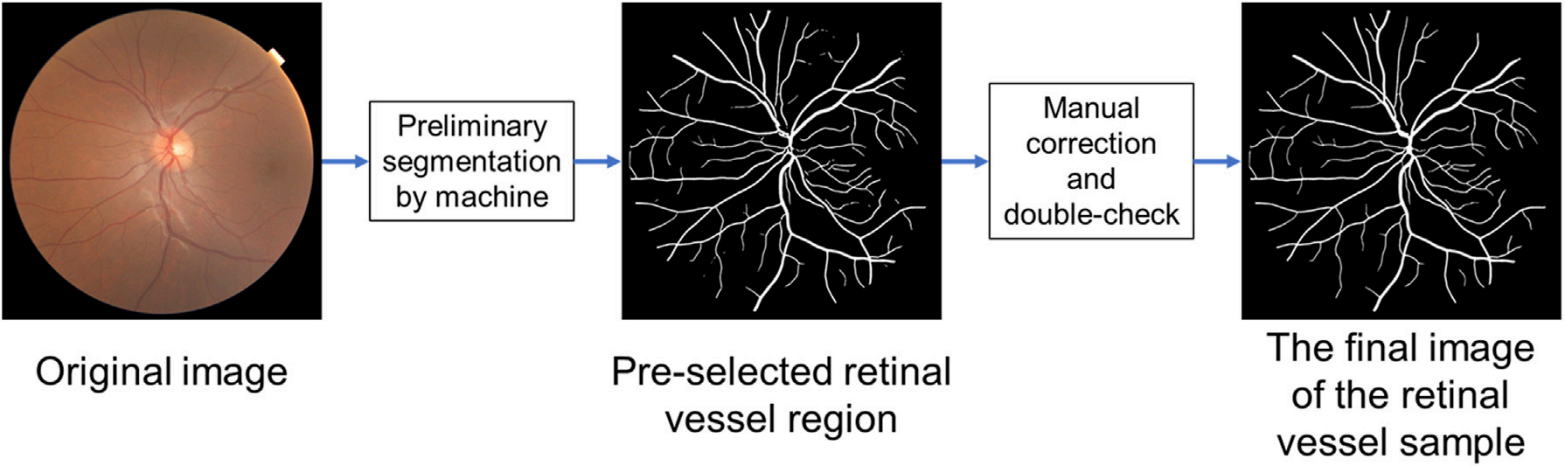
The process of labeling fundus photographs.

And then the resulting grayscale image is segmented using the Otsu algorithm to obtain the dark region. As a next step, the segmented dark region is filtered based on the brightness and morphology of the blood vessels on the fundus image to obtain the preselected blood vessel region. For the extracted retinal vascular regions, we used a trained deep learning semantic segmentation model to distinguish between arteries and veins. Arterial vessels and venous vessels are distinguished and identified by the corresponding vessel color and brightness, as well as the connection and topological relationship between the vessels. Two senior attending ophthalmologists then manually corrected the segmentation, with one performing the initial correction and the other reviewing and performing any additional correction, to obtain the final image of the blood vessel sample (Figures 3, 4).

The labeled 755 case samples were divided into training and validation sets at a ratio of 655:100. The training set is input into the ResNet101-UNet network for model training, and the loss value of the network model is calculated with the validation set. The model parameters are adjusted and optimized according to the loss value. The training is stopped when the loss value no longer decreases to obtain the final vessel segmentation model. This model is then used to segment the blood vessels (Figure 2).

### Optic disc segmentation

**FIGURE 5:**
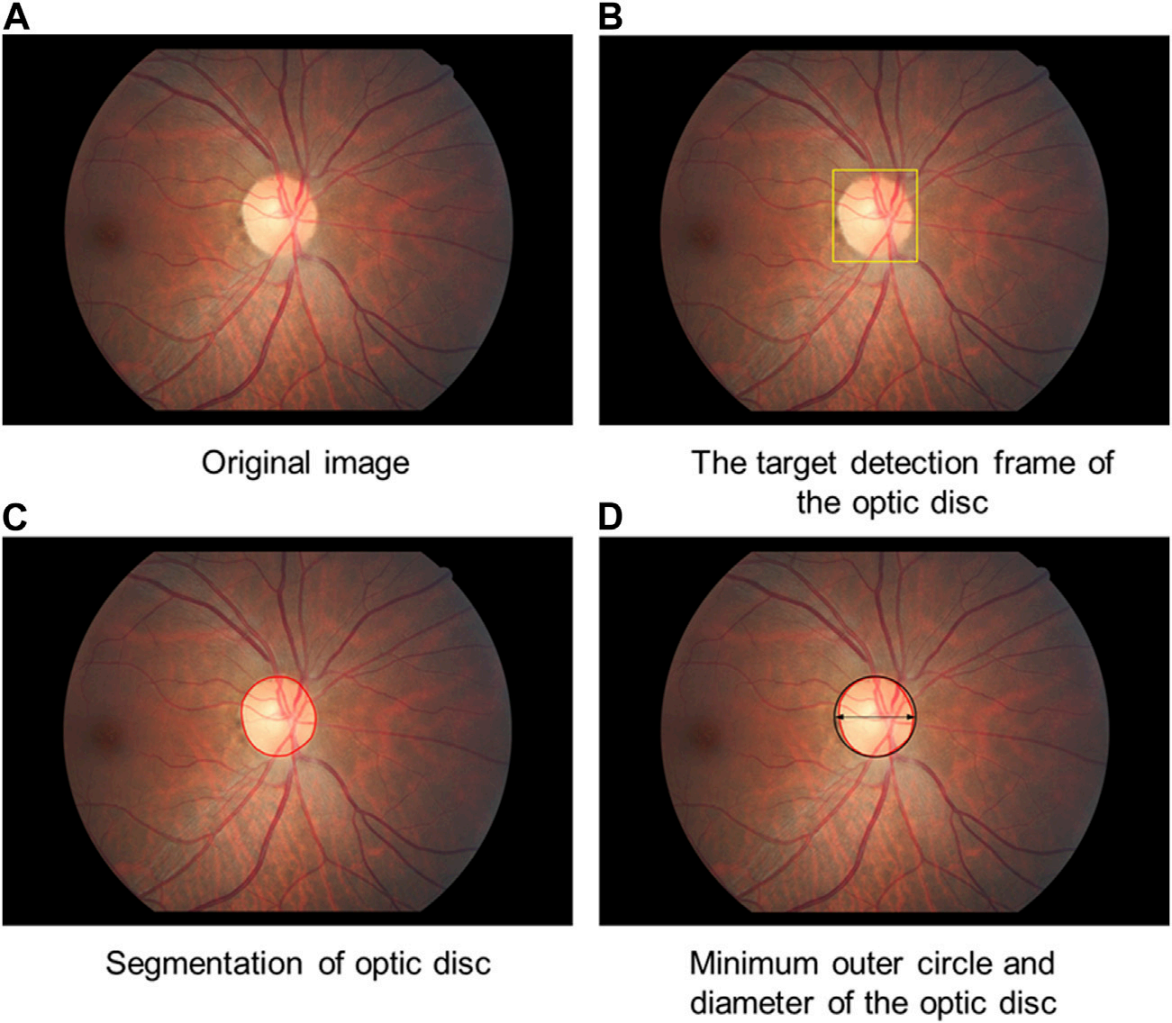
Diagram of optic disc segmentation. **(A)** Original image **(B)**: The target detection frame of the optic disc is obtained. **(C)** The boundary of the optic disc is determined. **(D)** The minimum outer circle is fitted to the segmented optic disc area.

In this study, optic disc segmentation was divided into two steps. First, the optic disc is detected using the deep learning object detection method to determine its location. The object detection model consists of single shot detection (SSD), and the backbone of the network structure is ResNet50. The model is trained with 2,000 training samples from the publicly available online Kaggle competition dataset to obtain the optic disc detection model and to ultimately obtain the object detection frame of the optic disc. The center point of the object detection frame is used as the center point of the optic disc. Afterward, the boundary of the optic disc is determined based on the visual attention mechanism. The center point determined by optic disc localization is used as the origin for performing a polar coordinate transformation on the fundus image. On the polar coordinate image, the edge detection operator is employed to determine the edge of the optic disc in polar coordinates. Then, the image is inversely transformed to finally obtain the edge of the optic disc in the image coordinate system to achieve detailed segmentation of the optic disc. Finally, the minimum outer circle is fitted to the segmented optic disc area. The center of the outer circle is used to locate the center point of the final optic disc, and the diameter of the optic disc is defined by the diameter of the outer circle (Figure 5).

### Accuracy evaluation of retinal vessel segmentation and optic disc segmentation

A sample of 100 manually annotated and reviewed color fundus images was used for the test, with each fundus image labeled separately for the retinal vessels and optic disc. Each manually annotated image was reviewed and corrected by two physicians, one for initial annotation or correction and the other for review and further correction. The automatic segmentation results of the model were compared with the manual annotation results. In units of pixels, accuracy (Acc), sensitivity, specificity, intersection over union (IoU), and DICE coefficients were calculated according to the following formulas. The results are shown in Table 1.

$$Acc=\frac{TP+TN}{TP+TN+FP+FN}$$

$$Sensitivity=\frac{TP}{TP+FN}$$

$$Specificity=\frac{TN}{TN+FP}$$

$$IoU=\frac{TP}{TP+FP+FN}$$

$$DICE=\frac{2\times TP}{FP+2\times TP+FN}$$

**TABLE 1:** Results of retinal vascular and optic disc segmentation accuracy evaluation.

Category	Accuracy (Acc)	Sensitivity	Specificity	Intersection ratio (IoU)	DICE
Retinal vascular segmentation	0.966	0.888	0.974	0.711	0.832
Optic disc segmentation	0.998	0.969	0.999	0.939	0.972

Note:

TP (True Positives): The prediction indicates that the sample is positive, and the prediction is accurate.

TN (True Negatives): The prediction indicates that the sample is negative, and the prediction is accurate.

FP (False Positives): The prediction indicates that the sample is positive, but the prediction is incorrect.

FN (False Negatives): The prediction indicates that the sample is negative, but the prediction is incorrect.

### Calculation of vascular fractal dimension

This study focuses on calculating the vascular fractal dimension. The fractal dimension was calculated on the segmented images. The fundus images were divided into several grids with different edge lengths (ε). In the grid corresponding to each edge length, the number of grid boxes intersecting retinal vessels was calculated as (N). The number of grid boxes intersecting with retinal vessels in each case and the inverse of its side length was fitted to a straight line in logarithm form, and the resulting slope of the line was the fractal dimension, which is calculated as follows.

$$\dim_{box}=\lim_{\varepsilon \to 0}\frac{logN\left(\varepsilon \right)}{\log\left(1/\varepsilon \right)}$$

### Calculation of retinal vascular density

The retinal vascular density is a quantitative representation of the state and coverage of fundus blood flow and has important clinical significance for detecting the occurrence, progression, and diagnosis of fundus diseases. Retinal vascular density refers to the retinal vascular area per unit fundus area; that is, in a certain area, the ratio of the area of the retinal vasculature to the area of the fundus on the photograph can be expressed as:

$$\rho =\frac{S^{\prime }}{S}$$

where *S′* is the extraction area of the retinal vasculature and *S* is the area of the fundus.

### Measurement of retinal vascular average diameter

**FIGURE 6:**
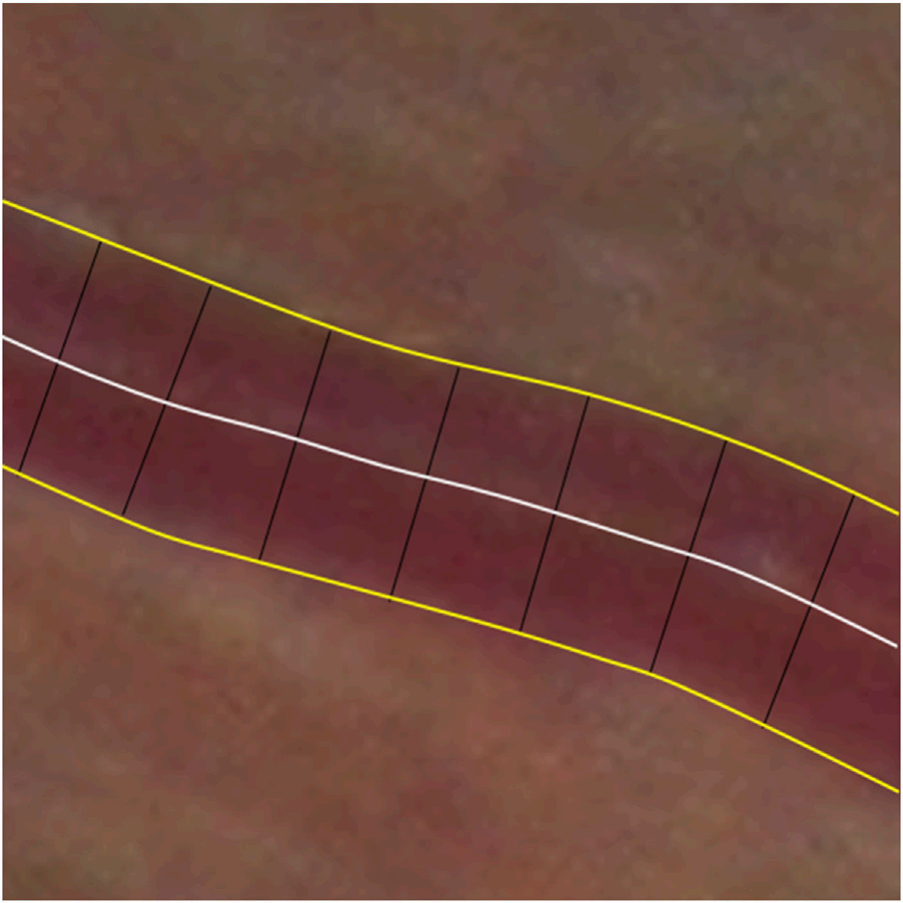
Schematic diagram of vascular diameter measurement.

Using the retinal vessel image obtained by segmentation, a bidirectional morphological erosion operation was applied based on the vessel boundary to determine the vessel centerline. At a certain step interval along the vessel centerline, the straight line orthogonal to the tangent line to a point on the centerline could be found. The orthogonal straight line intersects the vessel boundary at two points. The Euclidean distance *d* between those two points was calculated, and *d* is the vascular diameter corresponding to the point. The vessel diameter is calculated at 5-pixel intervals in the vessel direction The average retinal vascular diameter refers to the average of the vascular diameters corresponding to the points on the centerline. With the center of the optic disc as the reference origin and the diameter of the optic disc as the reference distance, the average value of the vascular diameter corresponding to the points on the centerline of the vessels in different regions was calculated, which was taken as the average vascular diameter value of the region. Finally, the optic disc diameter of 1.5 mm was used as a reference for unit conversion of the vascular diameter values (Figure 6).

### Measurement of retinal vascular tortuosity

The curvature corresponding to each point on the centerline of the vessel was calculated based on the following formula.

**FIGURE 7:**
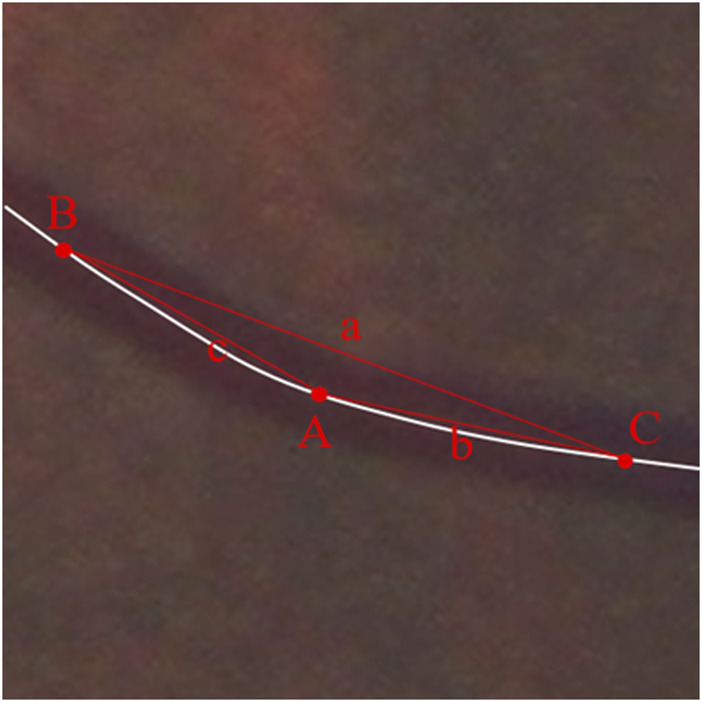
Schematic diagram of vascular tortuosity measurement. The curvature of each retinal vessel at a certain interval was measured and the average curvature to the points on the centerline of the vessel was calculated as the vascular average tortuosity.

Points B and C are identified on either side of point A such that their distances on the line from point A are equal, i.e., $\overset{⌢}{AB}=\overset{⌢}{AC}\cdot R_{A}$ is the radius of the external circle of △ABC composed of points A, B, and C, and $C_{A}$ is the curvature of the vessel at point A (Figure 7).

$$R_{A}=\frac{a}{2\sin A}$$

$$C_{A}=\frac{1}{R_{A}}$$

The curvature is calculated for each point on the vessel except for 25 pixels at the ends of the vessel (as the conditions for calculation are not met). The average curvature to the points on the centerline of the vessel was calculated as the vascular average tortuosity. Retinal vascular tortuosity was calculated as the average of the curvatures of each retinal vessel at a certain interval for all the extracted retinal vessels. Using the center of the optic disc as the reference origin and the diameter of the optic disc as the reference distance, the average tortuosity in different regions (C1, C2, C3, and C4 as mentioned above) was calculated (Figure 7).

### Measurement of retinal vascular branching angle

**FIGURE 8:**
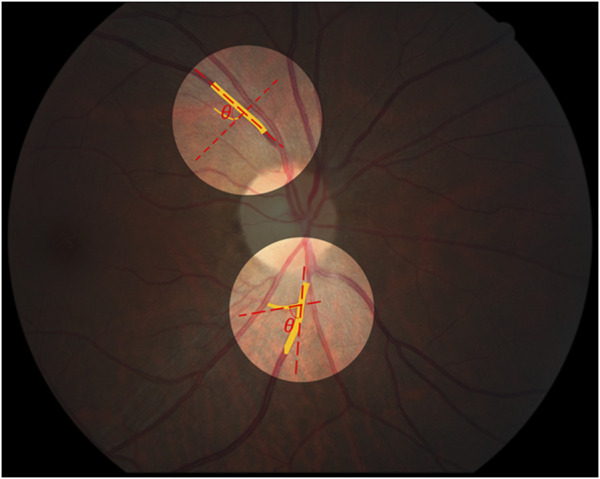
Schematic diagram of vascular branch angle measurement.

In this study, the branching angle of the vessel was calculated as the average angle between the main vessel and the branch vessels. Within a distance of 2 PD from the optic disc boundary, the retina was divided into upper and lower halves using the optic disc as a reference, and the vessel with the largest diameter was taken as the main vessel in each half. Based on the centerline of the vessel, the number of neighboring pixels corresponding to each pixel point on the centerline of the main vessel was calculated based on the 8-neighborhood algorithm, and the point with three neighboring pixels was selected as the branching point. Using the branching point as the starting point, a point 10 pixels away from the branching point was extracted from the centerline of the main vessel and the centerline of the corresponding branch vessel, and a straight line was fitted to each of them, after which the angle between the two lines was measured and calculated as the angle at the branching point. All the angles on the main vessel within 2 PD from the optic disc boundary were obtained, and the average value of all the angles were calculated and output as the retinal vascular branching angle (Figure 8).

### Statistical analysis

Statistical analyses were performed in Statistical Package for Social Science (SPSS, version 25.0, IBM Corp., Armonk, New York, United States) and GraphPad Prism 9.4.0 (GraphPad Software, San Diego, CA, United States). Population summary measures and retinal vascular parameters are described using the mean and SD for continuous variables or frequencies and percentages for categorical variables. Independent samples *t*-tests and chi-squared tests were used to compare the differences in participant characteristics between participants with and without cognitive impairment. The Kruskal‒Wallis test was used to determine the differences in retinal vascular parameters in the whole area and four annular zones among the four groups. Then, *post hoc* multiple comparisons (Bonferroni correction) were performed to determine pairwise differences. Furthermore, we included a multivariate linear regression analysis to test associations between the MMSE score and retinal vascular parameters. The MMSE score was defined as a dependent parameter, and the parameters that were significantly associated with MMSE were appropriately selected as independent parameters. All *p* values were two-sided, and *p* < 0.05 indicated statistical significance. Ninety-five percent confidence intervals are presented.

## Results

### Demographic characteristics

Among the 3,468 participants, 3,107 individuals (89.6%) [1,351 (43.5%) male] for whom fundus photographs centered on the optic disc and measurements of cognitive function were available were included in the current study. Participants had a mean age of 64.18 ± 9.75 years (median: 63 years; range: 50–93 years) and 74.1% had an education level classified as middle school education or college or higher education. The characteristics of the study population are summarized in detail in Table 2. Participants with cognitive impairment were more likely to be older, female, and a current smoker and to have lower BCVA (logMAR), less education, a higher BMI, and a higher prevalence of hypertension than participants without cognitive impairment.

**TABLE 2:** Participant summary characteristics.

Patient characteristics	ALL (*n* = 3107)	Normal cognition (*n* = 2693)	Cognitive impairment (*n* = 414)	*p*-value
Age (years, Mean ± SD)	64.18 ± 9.75	63.41 ± 9.37	69.15 ± 10.69	**<0.001**
Male, n (%)	1,351 (43.5%)	1,235 (45.9%)	116 (28.0%)	**<0.001**
BCVA (logMAR) (Mean ± SD)	0.94 ± 0.22	0.97 ± 0.19	0.76 ± 0.27	**<0.001**
Education, middle school, college or higher, n (%)	2,216 (74.1%)	2,172 (83.5%)	44 (11.3%)	**<0.001**
Currently smoking, yes, n (%)	628 (21.0%)	530 (20.4%)	98 (25.3%)	**0.027**
BMI (kg/m2, Mean ± SD)	25.59 ± 3.85	25.53 ± 3.77	26.01 ± 4.30	**0.035**
Hypertension, yes, n (%)	1,426 (50.7%)	1,190 (48.6%)	236 (64.8%)	**<0.001**
Diabetes, yes, n (%)	362 (13.8%)	322 (13.9%)	40 (12.9%)	0.644
Cardiovascular disease, yes, n (%)	507 (18.3%)	434 (17.8%)	73 (21.2%)	0.128
Infarction, yes, n (%)	204 (6.9%)	172 (6.7%)	32 (8.6%)	0.173
MMSE score (Mean ± SD)	26.36 ± 3.64	27.43 ± 1.98	19.36 ± 4.21	**<0.001**

Values are n (%) for categorical variables and mean ± SD for continuous variables. *p* values were calculated by independent samples t and chi-squared tests.

Abbreviations: BCVA, best corrected visual acuity; BMI, body mass index; MMSE, mini–mental state examination; SD, standard deviation.

*p* < 0.05 was considered statistically significant.

The bold values are to highlight *p* < 0.05.

### Retinal vascular characteristics in participants with different cognitive functions

The mean MMSE score was 26.34 ± 3.64 (median: 27; range: 2–30). Out of the 3,107 study participants, 414 (13.3%) individuals were classified as having cognitive impairment with an MMSE score < 24. A total of 296 (9.5%) of these individuals were classified as having mild cognitive impairment (MMSE score range: 19–23), 98 (3.2%) individuals were classified as having moderate cognitive impairment (MMSE score range: 10–18), and 20 (0.6%) individuals were classified as having severe cognitive impairment (MMSE score < 10).

Retinal vascular characteristics of the participants with different cognitive functions are detailed in Table 3. Between the normal cognition group and the mild cognitive impairment group, significant differences existed in retinal vascular average diameter (*p* < 0.001), retinal venular average diameter (*p* = 0.013), retinal vascular fractal dimension (*p* < 0.001), retinal vascular tortuosity (*p* < 0.001), and retinal vascular density (*p* < 0.001). Comparison of the different stages of cognitive impairment showed there were significant differences in the arteriole-to-venular ratio (mild cognitive impairment vs. severe cognitive impairment, *p* = 0.003; moderate cognitive impairment vs. severe cognitive impairment, *p* = 0.036), retinal vascular fractal dimension (mild cognitive impairment vs. moderate cognitive impairment, *p* = 0.001; mild cognitive impairment vs. severe cognitive impairment, *p* = 0.033), and retinal vascular density (mild cognitive impairment vs. moderate cognitive impairment, *p* = 0.001).

**TABLE 3:** Retinal vascular characteristics stratified by cognitive function.

Characteristics	All (N = 3107)	Normal cognition (*n* = 2693, 86.7%)	Mild cognitive impairment (*n* = 296, 9.5%)	Moderate cognitive impairment (*n* = 98, 3.2%)	Severe cognitive impairment (*n* = 20, 0.6%)	*p*-value	*Post hoc* comparisons	*Post hoc p*-value
Retinal vascular average diameter (um), mean ± SD	59.703 ± 4.457	59.475 ± 4.100	60.890 ± 5.871	61.697 ± 6.542	64.120 ± 7.396	**<0.001**	Normal cognition vs. Mild cognitive impairment	<0.001
							Normal cognition vs. Moderate cognitive impairment	0.001
							Normal cognition vs. Severe cognitive impairment	0.038
Retinal arteriolar average diameter (um), mean ± SD	49.348 ± 3.798	49.314 ± 3.601	49.527 ± 5.091	49.898 ± 4.493	48.843 ± 4.537	0.246	—	
Retinal venular average diameter (um), mean ± SD	70.359 ± 5.834	70.157 ± 5.577	71.373 ± 6.784	71.887 ± 8.121	75.858 ± 7.534	**<0.001**	Normal cognition vs. Mild cognitive impairment	0.013
							Normal cognition vs. Severe cognitive impairment	0.006
Arteriole-to-venular ratio, mean ± SD	0.704 ± 0.059	0.705 ± 0.056	0.698 ± 0.073	0.694 ± 0.072	0.648 ± 0.071	**<0.001**	Normal cognition vs. Severe cognitive impairment	0.001
							Mild cognitive impairment vs. Severe cognitive impairment	0.003
							Moderate cognitive impairment vs. Severe cognitive impairment	0.036
Retinal vascular fractal dimension, mean ± SD	1.512 ± 0.098	1.521 ± 0.074	1.471 ± 0.166	1.434 ± 0.206	1.401 ± 0.242	**<0.001**	Normal cognition vs. Mild cognitive impairment	<0.001
							Normal cognition vs. Moderate	<0.001
							Normal cognition vs. Severe	<0.001
							Mild cognitive impairment vs. Moderate cognitive impairment	0.001
							Mild cognitive impairment vs. Severe cognitive impairment	0.033
Retinal vascular branching angle (°), mean ± SD	55.484 ± 9.457	55.646 ± 9.458	54.660 ± 9.049	53.311 ± 10.129	54.496 ± 10.634	0.058	—	
^a^Retinal vascular tortuosity, mean ± SD	0.794 ± 0.163	0.788 ± 0.160	0.840 ± 0.173	0.797 ± 0.187	0.881 ± 0.213	**<0.001**	Normal cognition vs. Mild cognitive impairment	<0.001
							Mild cognitive impairment vs. Moderate cognitive impairment	0.047
Retinal vascular density, mean ± SD	0.086 ± 0.020	0.088 ± 0.018	0.079 ± 0.027	0.071 ± 0.026	0.067 ± 0.029	**<0.001**	Normal cognition vs. Mild cognitive impairment	<0.001
							Normal cognition vs. Moderate cognitive impairment	<0.001
							Normal cognition vs. Severe cognitive impairment	0.001
							Mild cognitive impairment vs. Moderate cognitive impairment	0.001

Values are n (%) for categorical variables and mean ± SD for continuous variables. *p* values were calculated by the Kruskal–Wallis test.

Abbreviations: SD, standard deviation.

^a^ Tortuosity values were multiplied by 1000 to be shown in Table.

*p* < 0.05 was considered statistically significant.

The bold values are to highlight *p* < 0.05.

To further analyses the relationship between retinal vascular parameters and cognitive function in individuals with different sexes, ages and hypertensive status, we conducted subgroup analyses to assess the relationship between retinal vascular parameters and cognitive impairment in individuals with and without hypertension, in individuals of different sexes, and in individuals of different age groups. The results are described in detail in Tables 4–6, respectively.

**TABLE 4:** Retinal vascular characteristics stratified by cognitive function in individuals with and without hypertension.

Characteristics	All (*n* = 3107)	Normal cognition (*n* = 2693, 86.7%)	Mild cognitive impairment (*n* = 296, 9.5%)	Moderate cognitive impairment (*n* = 98, 3.2%)	Severe cognitive impairment (*n* = 20, 0.6%)	*p*-value
With hypertension	*n* = 1426	*n* = 1190, 83.5%	*n* = 176, 12.3%	*n* = 49, 3.4%	*n* = 11, 0.8%	
Retinal vascular average diameter (um), mean ± SD	59.860 ± 4.638	59.527 ± 4.253	61.125 ± 5.758	62.649 ± 6.137	64.807 ± 8.099	**<0.001**
Retinal arteriolar average diameter (um), mean ± SD	49.074 ± 3.926	49.043 ± 3.664	49.099 ± 5.239	49.936 ± 4.573	48.310 ± 5.307	0.171
Retinal venular average diameter (um), mean ± SD	70.625 ± 6.083	70.343 ± 5.803	71.581 ± 7.006	73.105 ± 7.583	76.247 ± 7.691	**0.001**
Arteriole-to-venular ratio, mean ± SD	0.698 ± 0.060	0.700 ± 0.056	0.688 ± 0.077	0.688 ± 0.080	0.637 ± 0.078	**0.014**
Retinal vascular fractal dimension, mean ± SD	1.502 ± 0.109	1.512 ± 0.081	1.456 ± 0.187	1.461 ± 0.144	1.338 ± 0.313	**<0.001**
Retinal vascular branching angle (°), mean ± SD	55.540 ± 9.568	55.691 ± 9.571	54.587 ± 8.879	55.058 ± 11.135	54.524 ± 12.958	0.485
^a^Retinal vascular tortuosity, mean ± SD	0.809 ± 0.170	0.801 ± 0.168	0.857 ± 0.183	0.828 ± 0.165	0.844 ± 0.179	**<0.001**
Retinal vascular density, mean ± SD	0.083 ± 0.021	0.085 ± 0.019	0.077 ± 0.028	0.072 ± 0.023	0.062 ± 0.033	**<0.001**
Without hypertension	*n* = 1,389	*n* = 1,261, 90.8%	*n* = 90, 6.5%	*n* = 32, 2.3%	*n* = 6, 0.4%	
Retinal vascular average diameter (um), mean ± SD	59.549 ± 4.110	59.431 ± 3.954	60.995 ± 4.945	59.591 ± 5.867	62.352 ± 6.990	**0.010**
Retinal arteriolar average diameter (um), mean ± SD	49.639 ± 3.594	49.565 ± 3.515	50.486 ± 4.363	50.051 ± 4.071	50.266 ± 3.930	0.072
Retinal venular average diameter (um), mean ± SD	70.076 ± 5.523	70.002 ± 5.330	71.030 ± 6.323	69.625 ± 8.846	73.740 ± 8.657	0.375
Arteriole-to-venular ratio, mean ± SD	0.711 ± 0.057	0.710 ± 0.056	0.715 ± 0.069	0.704 ± 0.057	0.686 ± 0.064	0.323
Retinal vascular fractal dimension, mean ± SD	1.525 ± 0.067	1.528 ± 0.059	1.501 ± 0.099	1.462 ± 0.162	1.514 ± 0.032	**<0.001**
Retinal vascular branching angle (°), mean ± SD	55.672 ± 9.361	55.814 ± 9.376	55.192 ± 9.237	51.322 ± 8.153	55.064 ± 10.638	0.067
^a^Retinal vascular tortuosity, mean ± SD	0.776 ± 0.153	0.773 ± 0.149	0.810 ± 0.158	0.756 ± 0.214	0.919 ± 0.260	**0.013**
Retinal vascular density, mean ± SD	0.090 ± 0.018	0.090 ± 0.017	0.084 ± 0.024	0.073 ± 0.025	0.084 ± 0.013	**<0.001**

Values are n (%) for categorical variables and mean ± SD for continuous variables. *p* values were calculated by the Kruskal–Wallis test.

Abbreviations: SD, standard deviation.

^a^ Tortuosity values were multiplied by 1000 in order to be shown in Table.

*p* < 0.05 was considered statistically significant.

The bold values are to highlight *p* < 0.05.

**TABLE 5:** Retinal vascular characteristics stratified by cognitive function in different sexes.

Characteristics	All (*n* = 3107)	Normal cognition (*n* = 2693, 86.7%)	Mild cognitive impairment (*n* = 296, 9.5%)	Moderate cognitive impairment (*n* = 98, 3.2%)	Severe cognitive impairment (*n* = 20, 0.6%)	*p*-value
Male	*n* = 1351	*n* = 1235, 91.4%	*n* = 81, 6.0%	*n* = 27, 2.0%	*n* = 8, 0.6%	
Retinal vascular average diameter (um), mean ± SD	59.786 ± 4.372	59.647 ± 4.203	60.571 ± 4.876	63.175 ± 6.883	63.185 ± 8.586	**0.008**
Retinal arteriolar average diameter (um), mean ± SD	49.223 ± 3.720	49.201 ± 3.620	49.728 ± 4.628	49.186 ± 4.702	47.731 ± 5.537	0.329
Retinal venular average diameter (um), mean ± SD	70.418 ± 5.650	70.312 ± 5.562	70.625 ± 5.499	74.154 ± 8.033	73.531 ± 8.367	0.104
Arteriole-to-venular ratio, mean ± SD	0.702 ± 0.056	0.702 ± 0.055	0.705 ± 0.058	0.669 ± 0.080	0.656 ± 0.101	**0.031**
Retinal vascular fractal dimension, mean ± SD	1.516 ± 0.080	1.521 ± 0.060	1.480 ± 0.129	1.386 ± 0.289	1.411 ± 0.158	**<0.001**
Retinal vascular branching angle (°), mean ± SD	56.347 ± 8.983	56.361 ± 8.953	56.433 ± 9.261	54.739 ± 8.774	58.795 ± 13.472	0.904
^a^Retinal vascular tortuosity, mean ± SD	0.784 ± 0.151	0.781 ± 0.148	0.832 ± 0.180	0.769 ± 0.155	0.816 ± 0.170	**0.027**
Retinal vascular density, mean ± SD	0.086 ± 0.019	0.087 ± 0.018	0.078 ± 0.025	0.069 ± 0.024	0.059 ± 0.033	**<0.001**
Female	*n* = 1756	*n* = 1458, 83.0%	*n* = 215, 12.2%	*n* = 71, 4.0%	*n* = 12, 0.7%	
Retinal vascular average diameter (um), mean ± SD	59.639 ± 4.522	59.327 ± 4.006	61.010 ± 6.212	61.175 ± 6.387	64.799 ± 6.755	**<0.001**
Retinal arteriolar average diameter (um), mean ± SD	49.446 ± 3.856	49.411 ± 3.583	49.449 ± 5.267	50.161 ± 4.421	49.652 ± 3.727	0.427
Retinal venular average diameter (um), mean ± SD	70.313 ± 5.974	70.026 ± 5.587	71.656 ± 7.204	71.088 ± 8.058	77.551 ± 6.758	**<0.001**
Arteriole-to-venular ratio, mean ± SD	0.706 ± 0.060	0.708 ± 0.057	0.695 ± 0.078	0.703 ± 0.067	0.642 ± 0.043	**<0.001**
Retinal vascular fractal dimension, mean ± SD	1.510 ± 0.109	1.520 ± 0.085	1.468 ± 0.178	1.452 ± 0.164	1.394 ± 0.293	**<0.001**
Retinal vascular branching angle (°), mean ± SD	54.807 ± 9.762	55.032 ± 9.833	53.968 ± 8.894	52.775 ± 10.607	52.152 ± 8.547	0.120
^a^Retinal vascular tortuosity, mean ± SD	0.801 ± 0.172	0.794 ± 0.169	0.843 ± 0.170	0.806 ± 0.197	0.928 ± 0.237	**<0.001**
Retinal vascular density, mean ± SD	0.087 ± 0.021	0.088 ± 0.019	0.080 ± 0.028	0.071 ± 0.027	0.073 ± 0.025	**<0.001**

Values are n (%) for categorical variables and mean ± SD for continuous variables. *p* values were calculated by the Kruskal–Wallis test.

Abbreviations: SD, standard deviation.

^a^ Tortuosity values were multiplied by 1000 in order to be shown in Table.

*p* < 0.05 was considered statistically significant.

The bold values are to highlight *p* < 0.05.

**TABLE 6:** Retinal vascular characteristics stratified by cognitive function in different age groups.

Characteristics	All (*n* = 3107)	Normal cognition (*n* = 2693, 86.7%)	Mild cognitive impairment (*n* = 296, 9.5%)	Moderate cognitive impairment (*n* = 98, 3.2%)	Severe cognitive impairment (*n* = 20, 0.6%)	*p*-value
Age: 50–64 years	*n* = 1692	*n* = 1540, 91.0%	*n* = 127, 7.5%	*n* = 23, 1.4%	*n* = 2, 0.1%	
Retinal vascular average diameter (um), mean ± SD	58.743 ± 3.499	58.714 ± 3.443	59.179 ± 4.137	58.580 ± 3.422	55.784 ± 1.233	0.316
Retinal arteriolar average diameter (um), mean ± SD	48.950 ± 3.089	48.951 ± 3.066	49.149 ± 3.279	47.800 ± 3.509	48.397 ± 3.236	0.554
Retinal venular average diameter (um), mean ± SD	69.480 ± 4.934	69.446 ± 4.952	69.865 ± 4.700	70.073 ± 5.085	64.631 ± 0.453	0.158
Arteriole-to-venular ratio, mean ± SD	0.707 ± 0.051	0.707 ± 0.052	0.706 ± 0.048	0.683 ± 0.042	0.749 ± 0.055	0.120
Retinal vascular fractal dimension, mean ± SD	1.543 ± 0.046	1.544 ± 0.044	1.537 ± 0.070	1.537 ± 0.042	1.542 ± 0.012	<0.001
Retinal vascular branching angle (°), mean ± SD	55.730 ± 9.026	55.799 ± 9.065	55.152 ± 8.614	54.666 ± 8.751	50.626 ± 11.496	0.739
^a^Retinal vascular tortuosity, mean ± SD	0.805 ± 0.164	0.801 ± 0.162	0.841 ± 0.164	0.874 ± 0.248	0.756 ± 0.062	**0.011**
Retinal vascular density, mean ± SD	0.095 ± 0.013	0.095 ± 0.012	0.094 ± 0.017	0.092 ± 0.018	0.092 ± 0.003	0.736
Age: 65–79 years	*n* = 1188	*n* = 999, 84.1%	*n* = 137, 11.5%	*n* = 44, 3.7%	*n* = 8, 0.7%	
Retinal vascular average diameter (um), mean ± SD	60.671 ± 4.780	60.357 ± 4.528	62.212 ± 5.726	62.915 ± 5.702	62.460 ± 5.407	**<0.001**
Retinal arteriolar average diameter (um), mean ± SD	49.838 ± 4.349	49.768 ± 4.147	50.034 ± 5.620	50.971 ± 4.473	49.580 ± 4.185	0.146
Retinal venular average diameter (um), mean ± SD	71.239 ± 6.308	71.034 ± 6.119	72.073 ± 6.970	72.752 ± 8.020	74.997 ± 5.738	**0.044**
Arteriole-to-venular ratio, mean ± SD	0.702 ± 0.065	0.703 ± 0.061	0.697 ± 0.084	0.700 ± 0.083	0.663 ± 0.059	0.162
Retinal vascular fractal dimension, mean ± SD	1.489 ± 0.103	1.497 ± 0.083	1.446 ± 0.172	1.427 ± 0.167	1.499 ± 0.020	**<0.001**
Retinal vascular branching angle (°), mean ± SD	55.236 ± 9.869	55.384 ± 9.856	54.403 ± 9.059	53.644 ± 12.144	57.308 ± 11.674	0.569
^a^Retinal vascular tortuosity, mean ± SD	0.783 ± 0.161	0.774 ± 0.158	0.844 ± 0.175	0.777 ± 0.125	0.867 ± 0.241	**<0.001**
Retinal vascular density, mean ± SD	0.079 ± 0.021	0.080 ± 0.019	0.072 ± 0.027	0.064 ± 0.025	0.079 ± 0.010	**<0.001**
Age: ≥80 years	*n* = 227	*n* = 154, 67.8%	*n* = 32, 14.1%	*n* = 31, 13.7%	*n* = 10, 4.4%	
Retinal vascular average diameter (um), mean ± SD	61.904 ± 6.775	61.411 ± 5.337	62.182 ± 9.757	62.473 ± 8.682	67.447 ± 8.044	0.083
Retinal arteriolar average diameter (um), mean ± SD	49.812 ± 5.085	50.039 ± 4.311	48.860 ± 8.164	50.103 ± 4.802	48.287 ± 5.361	0.886
Retinal venular average diameter (um), mean ± SD	72.423 ± 8.019	71.639 ± 6.668	74.626 ± 10.879	72.111 ± 10.095	79.119 ± 7.393	**0.024**
Arteriole-to-venular ratio, mean ± SD	0.689 ± 0.073	0.697 ± 0.063	0.664 ± 0.100	0.694 ± 0.077	0.612 ± 0.060	**0.005**
Retinal vascular fractal dimension, mean ± SD	1.407 ± 0.198	1.441 ± 0.134	1.318 ± 0.260	1.364 ± 0.290	1.294 ± 0.313	**0.002**
Retinal vascular branching angle (°), mean ± SD	54.839 ± 10.512	55.798 ± 10.767	53.426 ± 11.209	51.689 ± 8.007	52.389 ± 9.990	0.174
^a^Retinal vascular tortuosity, mean ± SD	0.765 ± 0.158	0.747 ± 0.131	0.814 ± 0.199	0.762 ± 0.193	0.921 ± 0.213	**0.039**
Retinal vascular density, mean ± SD	0.062 ± 0.025	0.066 ± 0.023	0.048 ± 0.027	0.062 ± 0.022	0.051 ± 0.034	**0.004**

Values are n (%) for categorical variables and mean ± SD for continuous variables. *p* values were calculated by Kruskal–Wallis test.

Abbreviations: SD, standard deviation.

^a^ Tortuosity values were multiplied by 1000 to be shown in Table.

*p* < 0.05 was considered statistically significant.

The bold values are to highlight *p* < 0.05.

### Regional characteristics of retinal vascular alterations

**FIGURE 9:**
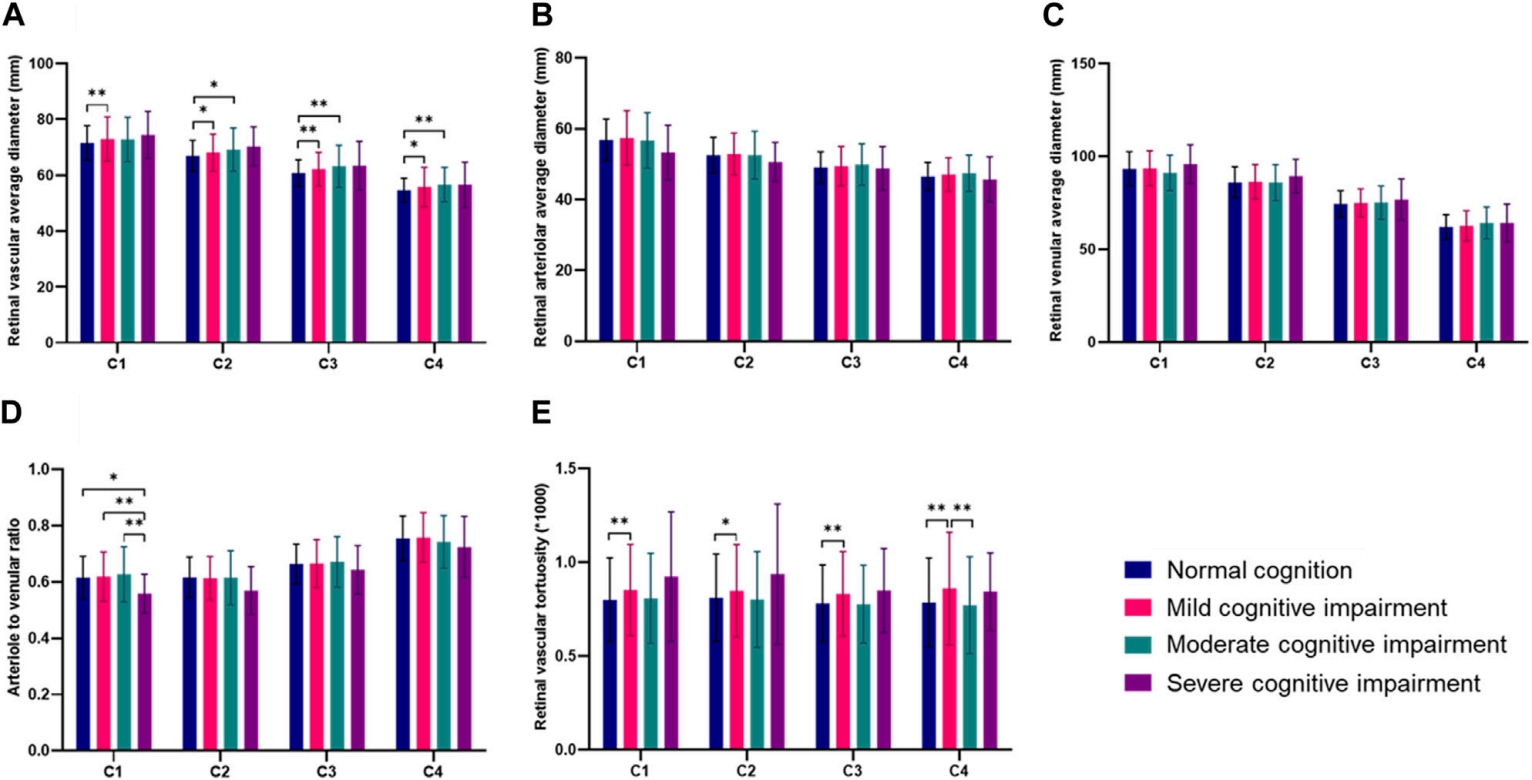
Comparisons of retinal vascular average diameter and retinal vascular tortuosity in four annular zones in different groups (*p < 0.05, **p < 0.01). **(A)** Retinal vascular average diameter in four annular zones in different groups. **(B)** Retinal arteriolar average diameter in four annular zones in different groups. **(C)** Retinal venular average diameter in four annular zones in different groups. **(D)** Arteriole to venular ratio in four annular zones in different groups. **(E)** Retinal vascular tortuosity in four annular zones in different groups.

The retinal vascular average diameter and retinal vascular tortuosity in four annular zones were further analyzed. Among the four annuli, the retinal vascular average diameter and retinal vascular tortuosity were significantly larger in the mild cognitive impairment group than in the normal cognition group (Figures 9A, E). In the C1 annuli, the arteriole-to-venular ratio was significantly lower in the severe cognitive impairment group than in the other three groups (Figure 9D). No significant differences in retinal arteriolar average diameter and venular average diameter were found within the four annuli (Figures 9B, C).

### Correlation between retinal vascular parameters and MMSE score

In the multivariate analysis, we first used the MMSE score as a dependent variable and all significantly associated systemic parameters (*p* < 0.05) as independent variables. Since the MMSE was affected by demographic factors, mostly by age and educational level, both parameters were included in the multivariate analysis in the present study (Jonas et al., 2018).

Second, we removed those parameters indicating a high degree of collinearity and parameters that were no longer significantly associated with the MMSE score (*p* > 0.05), including sex (*p* = 0.258), hypertension (*p* = 0.635), infarction (*p* = 0.708), current smoking (*p* = 0.417) and BMI (*p* = 0.121). After adjustment for age, BCVA (logMAR), and education level, a better MMSE score was significantly associated with a higher retinal vascular fractal dimension and higher retinal vascular density. For every 1 SD increase in the retinal vascular fractal dimension, the MMSE score increased by 0.134 points. (B = 0.134, 95% CI: 0.004∼0.263, *p* = 0.043). For every 1 SD increase in the retinal vascular density, the MMSE score increased by 0.152 points. (B = 0.152, 95% CI: 0.021∼0.283, *p* = 0.023) (Table 7).

**TABLE 7:** Associations (multivariate analysis) between the cognitive function score and retinal vascular parameters.

Parameter	Regression coefficient B	Standard error	Standardized coefficient beta	t	95% CI	*p* value
^a^Retinal vascular average diameter	−0.097	0.055	−0.027	−1.756	−0.206∼0.011	0.079
^a^Retinal arteriolar average diameter	−0.053	0.052	−0.015	−1.023	−0.156∼0.049	0.307
^a^Retinal venular average diameter	−0.022	0.053	−0.006	−0.416	−0.126∼0.082	0.677
^a^Arteriole-to-venular ratio	−0.032	0.052	−0.009	−0.619	−0.134∼0.070	0.536
^a^Retinal vascular fractal dimension	0.134	0.066	0.034	2.028	0.004∼0.263	**0.043**
^a^Retinal vascular branching angle	0.009	0.051	0.003	0.173	−0.091∼0.109	0.863
^a^Retinal vascular tortuosity	−0.048	0.052	−0.014	−0.925	−0.150∼0.054	0.355
^a^Retinal vascular density	0.152	0.067	0.042	2.282	0.021∼0.283	**0.023**

Abbreviations: CI, confidence interval.

^a^ The retinal vascular parameters were transformed into standardized Z-scores before inclusion in regression models.

Adjustment: age, education, best corrected visual acuity (logMAR).

*p* < 0.05 was considered statistically significant.

The bold values are to highlight *p* < 0.05.

## Discussion

In this study, we performed fully automated retinal vessel segmentation and quantitative measurement of retinal vascular parameters on color fundus photographs using artificial intelligence algorithms. With our fully automated quantitative results, this study demonstrated that alterations in retinal vascular parameters such as retinal vascular diameter, vascular fractal dimension, and vascular curvature are associated with cognitive impairment in a population-based cohort.

Previously, most of the quantitative measurements used for the analysis of retinal vascular parameters were semiautomated by computer-aided analysis programs, such as SIVA, IVAN, and VAMPIRE(Cheung et al., 2011b; Ong et al., 2013; McGrory et al., 2018; Mautuit et al., 2022). These methods require manual labeling and correction of the segmentation of the optic disc and retinal vessels. It takes a trained operator 20–30 min to correct the segmentation of each fundus image, and inevitably subjective bias will be introduced (Cheung et al., 2021b; Cheung et al., 2022). A variety of deep learning models for retinal vessel segmentation have emerged in recent years, and different deep learning models for vessel segmentation have sensitivities of approximately 0.72–0.95, specificities of 0.80–0.98, and accuracies of 0.91–0.98 (Chen et al., 2021). These studies, however, did not perform simultaneous quantitative measurements on the basis of vessel segmentation. Cheung et al. (2021b) developed a Singapore I Vessel Analyzer deep-learning system (SIVA-DLS) to automatically measure retinal vessel caliber. However, this system cannot detect other valuable retinal vascular parameters, such as vascular fractal dimension and retinal vascular tortuosity, at the same time. Shi et al. (2022) performed a meaningful study, in which they developed an artificial intelligence system for fully automated vessel segmentation and quantification of the retinal microvasculature. Sixteen basic parameters were included in the study. However, parameters such as vascular fractal dimension and vessel density, which are also of great importance, were not included. Wiseman et al. (2023) quantified retinal vessel density (VD) and branching complexity on optical coherence tomography angiography (OCTA). They found that retinal microvascular abnormalities exhibited on OCTA were associated with cerebral small vessel disease.

In this study, we constructed a vessel segmentation model based on the deep learning semantic segmentation network ResNet101-UNet, which can complete the process of optic disc segmentation and retinal vessel segmentation in a few seconds. This model is effective in reducing human error and has good sensitivity, specificity, and accuracy. In addition, based on retinal vessel segmentation, a fully automated detection method can then be used to quantitatively analyze the characteristics of the retinal microvascular system, deeply integrating deep learning and computer vision technologies to extract morphological information about all the vasculature in color fundus photographs, providing accurate measurements of retinal vascular branching angle, vascular fractal dimension, vascular average diameter, vascular average tortuosity, and other parameters.

Because age-related eye conditions (such as age-related macular degeneration and glaucoma) are common in older individuals, we intentionally included fundus photographs of patients with concomitant eye disease to increase applicability. Moreover, excluding eyes with these conditions may also introduce selection bias, as research indicates that cognitively impaired patients are more likely to have age-related macular degeneration and glaucoma (Lee et al., 2019; Cheung et al., 2021a).

In previous studies, research on the relationship between retinal vessel diameter and cognitive impairment has yielded mixed results. Liew et al. (2009) analyzed fundus photographs of the Blue Mountains Eye Study population and revealed that retinal venular dilation was associated with significant cognitive impairment, particularly in older persons with hypertension. In contrast, the Circulatory Risk in Communities Study reported that generalized arteriolar narrowing and a total number of retinal abnormalities may be useful markers for identifying persons at higher risk of disabling dementia (Jinnouchi et al., 2017). Recently, fully automated measurements of retinal arteriolar and venular calibers from retinal fundus images were estimated by Cheung et al. (2022) using a deep-learning system. They indicated that narrower retinal arteriolar caliber and wider retinal venular caliber are associated with an increased risk of cognitive decline. However, another study based on the Northern Ireland Cohort for the Longitudinal Study of Aging (NICOLA) showed that there were no associations between central retinal venular measures and mild cognitive impairment (O’Neill et al., 2021). The different findings may be due to inconsistent approaches to the definition of cognitive impairment or inconsistencies in the range of fundus photographs used to measure retinal vascular parameters.

Our study showed that the average retinal vascular diameter was increased in the cognitive impairment group compared to those with normal cognitive function. Separate analyses of retinal arteriolar average diameter and venular average diameter revealed that retinal arteriolar average diameter was not associated with cognitive impairment. In addition, the increase in retinal vascular average diameter mainly occurred in the venules. Evidence suggests that cognitive decline and related diseases may be associated with a wider retinal venular diameter (Yim-Lui Cheung et al., 2010; Simen et al., 2011), which is indicative of systemic inflammation, endothelial dysfunction, and abnormal blood-brain barriers (Nguyen and Wong, 2006; Wong et al., 2006).

In the present study, we found that retinal vascular fractal dimension and retinal vascular density may be more relevant candidate biomarkers for the early diagnosis of cognitive impairment than retinal vascular diameter. In individuals with cognitive impairment, the retinal vascular fractal dimension is decreased, and retinal vascular density is reduced. Similarly, Cheung et al. (2014) observed that Alzheimer’s disease (AD) patients had sparser retinal microvascular networks than controls, suggesting that retinal microvascular function is impaired in AD individuals. Ong et al. (2014) also indicated that reduced retinal arteriolar and venular fractal dimensions are associated with an increased risk of mild and moderate cognitive impairment. In a recent study, Xie et al. (2023) evaluated the association between changes in retinal microvasculature and Alzheimer’s disease and mild cognitive impairment using deep segmentation of OCTA images. They also revealed that the mild cognitive impairment group in their study had a reduced vascular fractal dimension compared with the control group. We performed a multiple linear regression analysis and found that retinal fractal dimension and retinal vascular density were positively correlated with the MMSE score. For every 1 SD increase in retinal fractal dimension, the MMSE increased by 0.134 points, and for every 1 SD increase in retinal vessel density, the MMSE increased by 0.152 points.

We analyzed the underlying reasons for the decrease in retinal vascular fractal dimension and retinal vascular density in a cognitively dysfunctional population. The fractal dimension of the retinal vasculature is an indicator of the complexity of the vascular branches, reflecting the distribution of blood throughout the retinal circulation, with larger values indicating a more complex distribution. When the fractal dimension of retinal vessels decreases, the sparse distribution of retinal vessels may represent the same alterations in the microvasculature of the brain (Nadal et al., 2020), indicating inadequate cerebral blood perfusion, which triggers activity in hypoxia-induced pathways that leads to pathological changes in tau, ultimately leading to the development and progression of cognitive impairment and even Alzheimer’s disease (Koike et al., 2011; Sabayan et al., 2012; Love and Miners, 2016).

We performed a *post hoc* test to compare pairs of the group with normal cognitive function and the groups with different stages of cognitive impairment. The results showed that there were significant differences in retinal venous diameter, vascular tortuosity, retinal vascular fractal dimension, and vascular density between the normal and mildly cognitively dysfunctional groups. On the other hand, between groups with different degrees of cognitive impairment, the severe cognitive impairment group had a reduced arteriole-to-venular ratio and a decreased retinal vascular fractal dimension compared to the mild and moderate cognitive impairment groups. These findings suggest that alterations in retinal venular average diameter, retinal vascular tortuosity, retinal vascular fractal dimension, and retinal vascular density may be early indicators of cognitive impairment, while reduced arteriole-to-venular ratio and decreased fractal dimension may be indicators of the progression of cognitive impairment.

We performed a zonation analysis on the retinal vascular parameters. Within the four annular regions C1-C4, the average vascular diameter, as well as the vascular tortuosity, were significantly different between the normal cognitive function group and the mild cognitive impairment group, suggesting that an increase in these two parameters within each region may occur in the early stages of cognitive impairment. Within the C1 zone, compared to that in the severe cognitive impairment group, the arteriole-to-venular ratio was significantly greater in the normal cognitive function group, the mild cognitive impairment group, and the moderate cognitive impairment group. The significant decrease in the arteriole-to-venular ratio in the severe cognitive impairment group suggested that the decrease in the arteriole-to-venular ratio of the central retinal vessels around the optic disc occurred late in the progression of cognitive impairment.

Our research has several advantages. First, computer intelligence-assisted measurement of vascular characteristics was used in this study to explore the relationship between retinal vascular characteristics and cognitive impairment based on the numerical indicators obtained. Compared with previous computer-aided detection of retinal vessels, the greatest breakthrough of this study is the automatic detection and segmentation of retinal vessels followed by the automatic calculation of vascular characteristic parameters, thus realizing a fully automated process of vascular parameter measurement. The fully automated process saves considerable time, reduces manpower costs, and avoids the subjective errors introduced by manual measurement or machine-assisted manual measurement. Second, we included data from population-based studies with large samples. In addition, we selected fundus images centered on the optic disc, which provide valuable information on the nasal vessels of the retina. Furthermore, we graded cognitive impairment and investigated the retinal vasculature in different regions to identify more accurate candidate biomarkers for early identification and late progression of cognitive impairment.

Potential limitations should be mentioned. First, it should be noted that 45° photographs of the central fundus were used for this study. The photographs included a limited area, which rendered it impossible to measure the peripheral vessels that are more susceptible to variations. Second, in the zonation analysis, we only performed the annular zones and did not divide them by quadrant, which may have yielded some meaningful results. We have only performed a zonal analysis of retinal vascular diameter and vascular tortuosity, without including retinal vascular fractal dimension and vascular density, which might have provided more meaningful information. Moreover, as this was a cross-sectional study, it was not possible to determine a potential causal relationship between the alterations in retinal vascular parameters and the decline in cognitive function.

In conclusion, we developed a vascular segmentation model based on deep learning algorithms to fully automate the quantitative measurement of retinal vascular parameters on fundus photographs. In this study, we demonstrated that an assessment of retinal vascular parameters with this model provided information on the risk of cognitive decline. The decrease in retinal vascular fractal dimension and decreased vascular density may serve as candidate biomarkers for early identification of cognitive impairment. The reduction in the retinal arteriole-to-venular ratio occurs in the late stages of cognitive impairment.

## Data Availability

The raw data supporting the conclusion of this article will be made available by the authors, without undue reservation.
